# Effect of plant growth-promoting *Streptomyces* sp. on growth promotion and grain yield in chickpea (*Cicer arietinum* L)

**DOI:** 10.1007/s13205-015-0283-8

**Published:** 2015-02-13

**Authors:** S. Gopalakrishnan, V. Srinivas, G. Alekhya, B. Prakash

**Affiliations:** International Crops Research Institute for the Semi-Arid Tropics (ICRISAT), Patancheru, 502 324 Telangana India

**Keywords:** Plant growth promotion, *Streptomyces* sp., Field demonstration, Scanning electron microscopy

## Abstract

The physiological and molecular responses of six strains of *Streptomyces* sp. (CAI-13, CAI-85, CAI-93, CAI-140, CAI-155 and KAI-180), with their proven potential for plant growth-promotion (PGP) in rice were studied to understand the mechanisms causing the beneficial effects. In this investigation, those six strains were evaluated for their PGP capabilities in chickpea in the 2012–13 and 2013–14 post-rainy seasons. All of the *Streptomyces* sp. strains exhibited enhanced nodule number, nodule weight, root weight and shoot weight at 30 days after sowing (DAS) and pod number, pod weight, leaf area, leaf weight and stem weight at 60 DAS in both seasons over the un-inoculated control. At chickpea crop maturity, the *Streptomyces* strains had enhanced stover yield, grain yield, total dry matter, pod weight, seed number and seed weight in both seasons over the un-inoculated control. In the rhizosphere, at crop maturity, the *Streptomyces* strains also significantly enhanced soil biological and mineral nutrient traits including microbial biomass carbon, dehydrogenase activity, total nitrogen, available phosphorous and organic carbon in both seasons over the un-inoculated control. Of the six strains of *Streptomyces* sp., CAI-85, CAI-93 and KAI-180 were found superior to CAI-155, CAI-140 and CAI-13, in terms of their effects on root and shoot development, nodule formation and crop productivity. Scanning electron microscopy micrographs had revealed the success in colonization of the chickpea roots by all six strains. This investigation further confirms the broad-spectrum of PGP activities by the selected *Streptomyces* sp.

## Introduction

Intensive farming practices, that warrant high yield and quality, require extensive use of chemical fertilizers and pesticides, which are costly and create environmental problems. Hence, there has been a resurgence of interest in environmental friendly, sustainable and eco-friendly agricultural practices (Esitken et al. 2002). Application of plant growth-promoting (PGP) bacteria is gaining importance in sustainable agricultural systems because of their low production costs, consumption of less non-renewable resources and eco-friendliness in nature. The mechanisms involved in plant growth promotion by PGP bacteria include secretion of PGP hormones, chelation of iron, nitrogen fixation, solubilization of phosphorus and inhibition of plant pathogens (Tokala et al. 2002; Soares et al. 2006; Cheng et al. 2007; Hao et al. 2011; Panhwar et al. 2012). Application of PGP bacteria on the rhizosphere had been reported to enhance root and shoot growth, nitrogen fixation and solubilization of minerals (Shaukat et al. 2006; Richardson et al. 2009). PGP bacteria such as *Rhizobium*, *Pseudomonas*, *Bacillus* and *Streptomyces* were reported to help the plants not only by mobilizing the nutrients but also by controlling plant pathogens (Postma et al. 2003; Perner et al. 2006; Gopalakrishnan et al. 2011a, b).

*Streptomyces*, a Gram positive bacterium with high G + C content, is the largest genus of actinomycetes having more than 500 species found predominantly in soil and decaying vegetation. They are known for PGP, breakdown of carbohydrates such as chitin and cellulose and degradation of soils contaminated with pesticides and heavy metals thereby playing a greater role in the process of phytoremediation (Glick 2010). PGP potential of *Streptomyces* was reported on cereals such as wheat (Sadeghi et al. 2012) and rice (Gopalakrishnan et al. 2013, 2014), legumes such as beans (Nassar et al. 2003) and peas (Tokala et al. 2002) and vegetables such as tomato (El-Tarabily 2008). *Streptomyces* promote plant growth by producing indole-3-acetic acid (IAA) (Aldesuquy et al. 1998) or siderophores (Tokala et al. 2002). Besides, *Streptomyces* has been extensively used for biocontrol of soil-borne fungal pathogens (Mahadevan and Crawford 1997; Trejo-Estrada et al. 1998; Macagnan et al. 2008; Gopalakrishnan et al. 2011b).

Chickpea (*Cicer arietinum* L) is the third most important pulse crop after beans (*Phaseolus vulgaris*) and peas (*Pisum sativum*) on a world basis but of utmost importance in the Mediterranean basin and south Asia, with a total production of 11.6 million tons from an area of 13.2 million ha and a productivity of 880 kg ha^−1^ (FAOSTAT 2011). Global yields of chickpea have been relatively stagnant for the last two decades (Millan et al. 2006; Rao et al. 2010). Conventional and molecular breeding approaches have made success stories in creating cultivars with improved resistance to various biotic and abiotic stresses. However, adoption of such improved cultivars in developing countries is still limited because of the fluctuating environmental factors and their consequences in yield parameters in crops (Ribaut et al. 2010; Tester and Langridge 2010; Varshney et al. 2012). Hence, in the present study, it was proposed to use PGP *Streptomyces* as an eco-friendly and sustainable tool to enhance the plant growth and yield of chickpea. Previously, we demonstrated a set of six *Streptomyces* strains (CAI-13, CAI-85, CAI-93, CAI-140, CAI-155 and KAI-180) isolated from herbal vermicompost, with a potential for PGP in rice (Gopalakrishnan et al. 2014). The objectives of this investigation were to further demonstrate the six *Streptomyces* strains for their PGP traits in chickpea under field conditions and to confirm the colonizing ability in chickpea by scanning electron microscopy (SEM) analysis.

## Materials and methods

Six strains of *Streptomyces*, CAI-13 (*Streptomyces caviscabies*; NCBI accession: KF770891), CAI-85 (*Streptomyces caviscabies*; NCBI accession: KF770897), CAI-93 (*Streptomyces globisporus*; NCBI accession: KF742498), CAI-140 (*Streptomyces griseorubens*; NCBI accession: KF742497), CAI-155 (*Streptomyces caviscabies*; NCBI accession: KF770896) and KAI-180 (*Streptomyces globisporus*; NCBI accession: KF742499), reported earlier by us as having potential for PGP in rice (Gopalakrishnan et al. 2014), were used in this study to prove their efficiency on plant growth promotion in chickpea.

The field trials were conducted for two cropping seasons in 2012–13 and 2013–14 at ICRISAT, Patancheru (17°30N; 78°16E; altitude 549 m) in peninsular India. The soil depth of the experimental site used was ≥1.2 m and this soil retained 200 mm of plant-available water in the 120 cm (maximum rooting depth) soil profile. The experimental field was kept fallow except for post-rainy season crop. The soil at the experimental site was Vertisols (fine montmorillonitic isohyperthermic typic pallustert) with 53 % clay, 25 % sand and 21 % silt with an alkaline pH of 7.6–8.3 and an organic carbon content of 0.4–0.6 %. The mineral content of the top 0–15 cm soils include 24.7, 8.6 and 298 mg kg^−1^ soil of available N, P and K, respectively. The fields were prepared into broad beds and furrows with 1.2 m wide beds flanked by 0.3-m furrows in both seasons (2012–13 and 2013–14). Surface application and incorporation of 18 kg N ha^−1^ and 20 kg P ha^−1^ as di-ammonium phosphate (DAP), were carried out 3 days before sowing in both seasons. The experiment was laid out with three replicates and subplot sizes of 4.0 × 3 m rows in ridges in a randomized complete block design (RCBD) under irrigated condition.

The *Streptomyces* strains (CAI-13, CAI-85, CAI-93, CAI-140, CAI-155 and KAI-180) were grown individually on a starch casein broth (starch—10 g; casein—0.3 g; sodium chloride—2 g; potassium nitrate—2 g; dipotassium hydrogen phosphate—2 g; magnesium sulfate—0.05 g; calcium carbonate—0.02 g; ferrous sulfate—0.01 g and; distilled water—1,000 ml) at 28 °C for 4–5 days. Seeds of chickpea variety ICCV 2 (duration 90 days) were treated individually with a *Streptomyces* strain (10^8^ CFU ml^−1^) for 45 min and hand sown on Oct 2012 in the first year and Nov 2013 in the second year with a spacing of 30 cm apart, and a depth of 5 cm to have an estimated plant stand of at least 26 plants m^−2^. *Streptomyces* strain (1,000 ml/replicate; 10^8^ CFU ml^−1^) was applied once in 15 days, from the sowing day, on the soil close to the plant until the flowering stage. Control plots contained no strains of *Streptomyces*. No serious plant pathogens or insect pest attacks were observed during the cropping period and the plots were kept weed-free by manual weeding. The plots were irrigated on 21 and 49 days after sowing (DAS). The chickpea crop was harvested manually on Jan 2013 in the first year and Feb 2014 in the second year. Samplings were done at 30 DAS, 60 DAS and at crop maturity. On the sampling day, plant underground and aerial parts were harvested separately with care to eliminate border effects in each plot. During both the seasons of 2012–13, 2013–14, chickpea growth performance was measured in terms of nodule number, nodule weight, root weight and shoot weight at 30 DAS; plant height, pod number, pod weight, leaf area, leaf weight and stem weight at 60 DAS; and stover yield, grain yield, total dry matter (TDM), 1000-seed weight, pod weight, seed number and seed weight at crop maturity. Also, at crop maturity, rhizosphere soil samples were collected from top 0 to 15 cm soil and analyzed for total nitrogen (Novozamsky et al. 1983), available phosphorous (Olsen and Sommers 1982), organic carbon (Nelson and Sommers 1982), microbial biomass carbon (fumigation method; Anderson and Domsch 1989) and dehydrogenase activity (triphenyl formazan production method; Casida 1977).

The colonizing capability of *Streptomyces* strains on the roots of chickpea was examined by scanning electron microscopy (SEM) analysis, as per the protocols of Bozzola and Russell (1998). In brief, the seeds of chickpea (variety ICCV 2) were surface-sterilized at first with 2.5 % sodium hypochlorite, followed by 70 % ethanol (for 5 min each) and rinsed thoroughly with sterilized water before being allowed to sprout in a Petri dish overnight. At the end of incubation, the sprouted seeds were soaked into test *Streptomyces* strains (CAI-13, CAI-85, CAI-93, CAI-140, CAI-155 and KAI-180; grown in starch casein broth separately) for 45 min before being sown in pots containing sterilized coarse sand (six seeds/8 in plastic pot). *Streptomyces* strains (10^8^ CFU ml^−1^; 5 ml per seedling), as booster doze, were applied upon germination of the seed by soil drench method. The pots were incubated at 24 ± 2 °C in a greenhouse for 2 weeks. After that, seedlings of chickpea were removed carefully from the pots and the roots were washed in 0.1 M phosphate buffer (pH 7.2). The tips of the roots were cut into 5 mm long pieces and fixed in glutaraldehyde (2.5 %) in phosphate buffer for 24 h at 4 °C. At the end of incubation, the root samples were again washed with phosphate buffer, postfixed in osmium tetroxide (2 %) for 4 h and dehydrated using a graded series of ethanol. The dehydrated samples were dried with critical-point liquid carbon dioxide as a transition fluid and adhered onto aluminum specimen mounted with double-stick adhesive tape. The mounted root samples were coated with gold–palladium in an automated sputter coater (JEOL JFC-1600) and examined under a scanning electron microscope (JOEL-JSM 5600) as per the standardized protocols at RUSKA lab, College of Veterinary Science, Rajendranagar, Hyderabad, India.

Data were analyzed by analysis of variance (ANOVA) and the GLM (General Linear Model) procedure in the software package SAS (SAS Inst. 2002–08, SAS V9.3) considering isolates and replication as fixed in RCBD. Isolate means were tested for significance and compared using Fisher’s protected least significant difference (LSD) test.

## Results and discussion

The six *Streptomyces* strains (CAI-13, CAI-85, CAI-93, CAI-140, CAI-155 and KAI-180) studied in this investigation were reported previously to have potential for PGP in rice (Gopalakrishnan et al. 2014). In the present investigation, these strains were evaluated for their PGP traits in chickpea under field conditions. The plots treated with *Streptomyces* strains showed significantly enhanced agronomic performance of all the traits measured at 30 and 60 DAS. As seen at 30 DAS in both 2012–13 and 2013–14 seasons, the *Streptomyces* treated plots showed significantly enhanced agronomic traits including the nodule numbers (35–83 %), nodule weight (27–64 %), root weight (9–14) and shoot weight (32–50 %) over the un-inoculated control (Table 1). Similarly at 60 DAS, the *Streptomyces* strains significantly enhanced the pod number (29–95 %), pod weight (30–135 %), leaf area (32–34 %), leaf weight (25–63 %) and stem weight (23–68 %) and at crop maturity, stover yield (25–75 %), grain yield (11–26 %), total dry matter (19–32), pod weight (6–51 %), seed number (20–52) and seed weight (3–47 %) in both 2012–13 and 2013–14 seasons over the un-inoculated control plots (Tables 1, 2). In the top 15 cm rhizosphere soils, at crop maturity, the *Streptomyces* strains treated plots significantly enhanced the microbial biomass carbon (35–55), dehydrogenase activity (27–56 %), total N (4–11), available P (51–106 %) and organic carbon (9–16 %) in both 2012–13 and 2013–14 seasons over the un-inoculated control plots (Table 3).

**Table 1:** Effect of the six *Streptomyces* sp. on agronomic performance of chickpea under field conditions––at 30 and 60 days after sowing

Isolate	30 Days after sowing								60 Days after sowing									
	Nodule number (plant^−1^)		Nodule weight (mg plant^−1^)		Root weight (mg plant^−1^)		Shoot weight (g plant^−1^)		Plant height (cm)		Stem weight (g plant^−1^)		Leaf area (cm^−2^ plant^−1^)		Leaf weight (g plant^−1^)		Pod number (plant^−1^)	
	Y1	Y2	Y1	Y2	Y1	Y2	Y1	Y2	Y1	Y2	Y1	Y2	Y1	Y2	Y1	Y2	Y1	Y2
CAI-85	14	62	42	280	187	191	2.02	2.27	52	50	5.24	4.78	714	836	4.52	4.64	71	76
CAI-93	16	65	34	266	181	175	1.84	1.98	53	49	4.85	3.96	896	672	5.71	3.95	84	64
CAI-13	22	50	55	223	180	171	1.61	1.76	50	48	4.59	3.98	705	720	5.28	3.78	63	59
CAI-140	20	60	81	228	183	181	1.49	1.93	51	48	3.90	4.22	709	743	4.82	3.94	71	73
CAI-155	22	52	63	229	173	169	1.56	1.94	51	48	3.40	3.96	690	712	4.43	4.15	54	69
KAI-180	22	50	33	247	179	190	1.51	1.87	55	50	5.63	4.05	851	717	6.99	4.05	84	59
Control	12	49	29	221	171	168	1.35	1.72	50	47	3.36	3.88	670	632	4.29	3.71	43	59
Mean	18	56	48	242	179	178	1.63	1.92	52	49	4.43	4.12	748	719	5.15	4.03	67	66
SE±	1.5**	0.9***	3.1***	9.4**	2.8*	4.6**	0.070***	0.066**	0.3***	0.5**	0.021***	0.178*	11.1***	16.2***	0.108***	0.157*	2.8***	2.5***
LSD (5 %)	4.7	2.8	9.4	28.9	8.5	14.1	0.217	0.204	1.0	1.6	0.065	0.549	34.1	49.9	0.333	0.484	8.5	7.8
CV %	14	3	11	7	3	4	8	6	1	2	1	8	3	4	4	8	7	7

*Y1* year 1 (2012–13), *Y2* year 2 (2013–14), *SE* standard error, *LSD* least significant differences, *CV* coefficients of variation

* Statistically significant at 0.05; ** Statistically significant at 0.05; *** Statistically significant at 0.001

**Table 2:** Effect of the six *Streptomyces* sp. on agronomic performance and yield potential of chickpea under field conditions—at harvest

Isolates	Stover yield (t ha^−1^)		Grain yield (t ha^−1^)		Total dry matter (t ha^−1^)		1000 seed (weight g)		Pod weight (g plant^−1^)		Seed number (plant^−1^)		Seed weight (g plant^−1^)	
	Y1	Y2	Y1	Y2	Y1	Y2	Y1	Y2	Y1	Y2	Y1	Y2	Y1	Y2
CAI-85	1.64	1.81	1.86	2.10	3.50	3.91	222	196	18.3	22.7	56	84	15.3	16.4
CAI-93	1.30	1.76	1.76	1.81	3.06	3.57	221	198	18.1	19.5	58	73	15.3	15.7
CAI-13	1.94	1.80	1.76	1.79	3.69	3.58	222	198	18.4	21.3	65	80	15.3	16.3
CAI-140	1.48	1.80	1.80	2.06	3.24	3.86	223	205	17.7	21.3	57	76	15.5	15.6
CAI-155	1.58	1.74	1.75	1.81	3.33	3.55	223	197	17.9	22.2	58	87	15.5	17.1
KAI-180	1.40	2.10	1.76	1.87	3.16	3.97	223	196	17.6	25.7	61	100	15.5	19.6
Control	1.11	1.68	1.68	1.67	2.79	3.34	220	195	17.4	17.0	54	66	15.0	13.3
Mean	1.49	1.81	1.77	1.87	3.26	3.68	222	198	17.9	21.4	58	81	15.4	16.3
SE±	0.049***	0.068*	0.011***	0.068**	0.047***	0.092**	0.4***	1.2***	0.18**	1.18**	1.2**	3.2***	0.08**	0.68**
LSD (5 %)	0.200	0.206	0.033	0.210	0.146	0.284	1.2	3.6	0.57	3.66	3.7	9.9	0.25	2.10
CV %	6	6	1	6	3	4	1	1	2	10	4	7	1	7

*Y1* year 1 (2012–13), *Y2* year 2 (2013–14), *SE* standard error, *LSD* least significant differences, *CV* coefficients of variation

* Statistically significant at 0.05; ** Statistically significant at 0.05; *** Statistically significant at 0.001

**Table 3:** Effect of the six *Streptomyces* sp. on rhizosphere soil microbial biomass carbon, dehydrogenase, total N, available P and organic carbon activities of chickpea under field conditions—at harvest

Isolates	Microbial biomass carbon (µg g^−1^ soil)		Dehydrogenase activity (µg TPF g^−1^ soil 24 h^−1^)		Total N (ppm)		Available P (ppm)		Organic carbon (%)	
	Y1	Y2	Y1	Y2	Y1	Y2	Y1	Y2	Y1	Y2
CAI-85	1,042	576	58.0	58.7	701	738	11.0	7.5	0.51	0.56
CAI-93	1,183	856	59.9	62.2	643	751	10.6	7.6	0.49	0.56
CAI-13	1,128	774	60.4	63.0	683	739	11.3	9.5	0.50	0.56
CAI-140	958	630	61.6	76.0	683	746	12.2	10.4	0.49	0.56
CAI-155	987	574	70.3	60.0	704	762	20.8	7.8	0.48	0.64
KAI-180	1097	822	63.1	86.7	637	755	19.3	8.8	0.49	0.60
Control	877	551	55.4	55.7	632	735	10.1	6.9	0.47	0.55
Mean	1039	683	61.2	66.1	669	746	13.6	8.3	0.49	0.57
SE±	20.7***	24.8***	2.13*	3.19**	11.8*	4.8*	0.18***	0.53*	0.004**	0.008**
LSD (5 %)	71.7	84.8	7.38	11.04	41.0	16.5	0.62	1.83	0.013	0.026
CV %	3	5	5	7	3	1	2	9	1	2

*Y1* year 1 (2012–13), *Y2* year 2 (2013–14), *SE* standard error, *LSD* least significant differences, *CV* coefficients of variation

* Statistically significant at 0.05; ** Statistically significant at 0.05; *** Statistically significant at 0.001

Of the six *Streptomyces* strains studied in the present investigation, CAI-85, CAI-93 and KAI-180 (in descending order) were found superior to CAI-155, CAI-140 and CAI-13 in terms of their effects on root and shoot development, nodule formation and crop productivity. Further, the six *Streptomyces* did not inhibit the growth of *Mesorhizobium ciceri* on yeast extract mannitol agar (YEMA; data not shown). Thus, it is concluded that the six *Streptomyces* strains are capable of enhancing agronomic and yield traits in chickpea and compatible with *Rhizobium*, so that co-inoculation of these two genera is possible. The effect of *Streptomyces* for PGP in crops including vegetables such as tomato, cereals such as wheat and rice and legumes such as beans and peas was reported widely (Tokala et al. 2002; Nassar et al. 2003; El-Tarabily 2008; Sadeghi et al. 2012; Gopalakrishnan et al. 2014). PGP bacteria with broad spectrum properties offer effective novel strategies not only for crop growth and yield but also for controlling insect pests and pathogens that attack crops. PGP bio-agents also bring forth induced systemic resistance against a broad range of pathogens and insect pests (Jetiyanon and Kloepper 2002; Ryu et al. 2007).

**Fig. 1:**
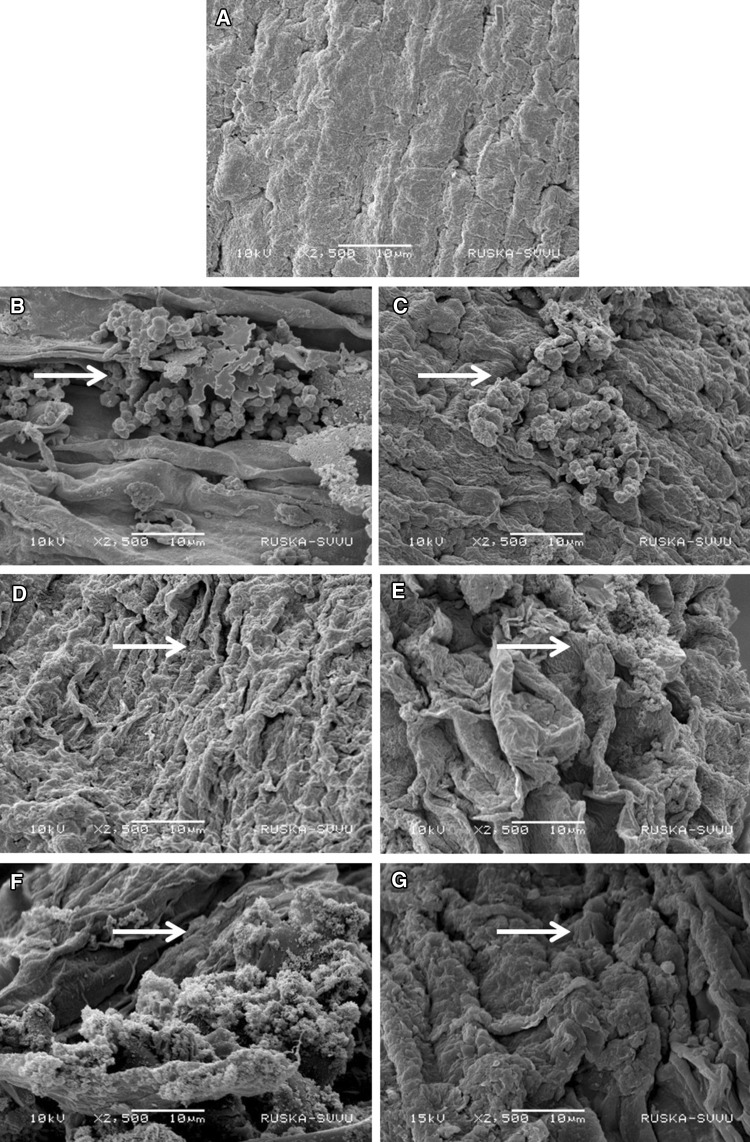
Scanning electron microscopy photographs of the six *Streptomyces* sp. showing colonization on the roots of chickpea. Representative SEM images of control and treated chickpea roots. **a** Normal surface of chickpea roots without any microbial treatment, **b**–**g** chickpea roots colonized by PGP *Streptomyces* strains CAI-13, CAI-85, CAI-93, CAI-140, CAI-155 and KAI-180 respectively

SEM analysis of chickpea roots showed a remarkable degree of colonization by all the six strains of *Streptomyces*. Roots from *Streptomyces* inoculated plants exhibited significant surface colonization while those from un-inoculated plants did not. Further, the sporulation of *Streptomyces* strains on the surface cell layer of chickpea roots was clearly evident for all six strains. The hyphae of *Streptomyces* strains were also found to penetrate the surface cell layer of chickpea roots (Fig. 1). Colonization by PGP bacteria at the right time and place is a pre-requisite for enhanced PGP activity. Host–bacteria interaction is important for colonization which involves sufficient population of bacteria, rhizosphere competence of the bacteria and root colonizing and PGP ability of the bacteria (Lugtenberg and Dekkers 1999). In the present investigation, SEM analysis demonstrated colonization of *Streptomyces* strains on the roots of chickpea. Therefore, the SEM analysis, in addition to the data for grain and stover yield, root and other agronomical traits, and the mineral nutrients and biological activities of the rhizosphere soil strongly suggest that the six *Streptomyces* strains had multiplied and colonized on the chickpea roots.

The mechanism by which the six *Streptomyces* strains consistently enhanced yield and agronomical traits on rice (from our previous study) and chickpea (from this study) could be attributed to their ability to produce siderophores, indole acetic acid (IAA) and β-1,3-glucanase activities (Gopalakrishnan et al. 2014). Bacteria are reported to have the ability to produce low-molecular weight siderophores that are capable of sequestering Fe^3+^ and to help plants to have their iron requirement. Siderophores act as solubilizing agents of iron from minerals under conditions of iron limitation (Indiragandhi et al. 2008). Siderophores are reported to form stable complexes with heavy metals such as Al, Cd, Cu, Ga, In, Np, Pb, U and Zn and increase the soluble metal concentration (Rajkumar et al. 2010). Therefore, siderophores help to alleviate the stresses imposed on plants by heavy metals in soils. IAA is the plant hormone that accelerates plant growth by enhancing shoot/root growth and seedling vigour. IAA synthesizing bacteria are known to stimulate seed germination, initiate root formation and increase root length and surface area thereby providing the host plant greater access to water and soil nutrients (Ahemad and Kibret 2014).The cell wall of plant pathogens, such as *Fusarium oxysporum* (causes wilt in many crops), contain layers of β-1,3-glucan and lysis of β-1,3-glucan layers by β-1,3-glucanase-producing bacteria leads to the leakage of cell contents and the collapse of the pathogenic fungi (Singh et al. 1999). Furthermore, qRT-PCR validation of IAA genes revealed that gene IAA showed higher up regulation in the CAI-85 followed by CAI-93 while gene siderophore was highly up-regulated in CAI-155 followed by CAI-85 and CAI-93 and this results confirmed the results of in vitro PGP attributes of the six *Streptomyces* strains (from our previous study; Gopalakrishnan et al. 2014).Hence, it is concluded that the *Streptomyces* strains used in this investigation apparently contained a broad range of PGP traits which can be exploited for PGP in cereal–legum cropping system.

It is concluded that the six *Streptomyces* strains (CAI-13, CAI-85, CAI-93, CAI-140, CAI-155 and KAI-180) studied in this investigation were apparently well adapted not only in rice rhizosphere, as reported earlier, but also in the chickpea rhizosphere. Among the six *Streptomyces* strains studied, CAI-85, CAI-93 and KAI-180 were found to be the best performers than other isolates in terms of agronomic performance and crop productivity. Therefore, these three strains are potential candidates for the discovery of novel secondary metabolites and their usefulness in integrated nutrient management programs that can help in furthering the use of eco-friendly bio-fertilizers. Further studies are needed to understand the importance of these six PGP *Streptomyces* in the rhizosphere and their potential use in the environment. Also, there is a need to do additional comprehensive research to exploit the potential of these PGP *Streptomyces* under different field conditions (multi-location trials), commercialization and improve sustainability in agricultural production.
